# An X-ray beam profile monitoring system at a beamline front-end combining a single-crystal diamond film and energy discrimination using droplet analysis

**DOI:** 10.1107/S1600577522002466

**Published:** 2022-04-20

**Authors:** Togo Kudo, Mutsumi Sano, Takahiro Matsumoto, Toshiro Itoga, Shunji Goto, Sunao Takahashi

**Affiliations:** a Japan Synchrotron Radiation Research Institute, 1-1-1 Sayo-cho, Sayo-gun, Hyogo 679-5198, Japan; b RIKEN SPring-8 Center (RSC), 1-1-1 Sayo-cho, Sayo-gun, Hyogo 679-5148, Japan

**Keywords:** X-ray beam profile monitor, pinhole, single-crystal diamond, front-end, energy discrimination

## Abstract

This work has successfully demonstrated a system for monitoring pink-beam X-rays exiting from a beamline front-end, which has a specific spatial distribution based on each energy component. In this study, the X-rays scattered from a single-crystal chemical-vapor-deposited diamond film were converted into a cross-sectional image using pinhole optics, followed by digitization with a direct detection complementary metal-oxide-semiconductor 2D detector. By using single crystals instead of poly-crystals, good quality images were obtained with no diffraction bright spots. As a result of applying photon energy discrimination using the droplet analysis to the image information, the spatial distribution of each energy component of the undulator radiation was successfully visualized. The result was found to be in good agreement with the theoretically calculated result obtained using the synchrotron radiation calculation code *SPECTRA*. The new synchrotron radiation beam monitor proposed in this paper can serve as a powerful beam diagnostic tool for diffraction-limited rings that require strict light source stability.

## Abstract 2

A beam monitoring system with pin-hole optics has been developed at an undulator beamline front-end in SPring-8. Energy-resolved beam images were successfully observed and were found to be in good agreement with the theoretical calculation.

## 1. Introduction

In diffraction-limited storage ring (DLSR) sources, a low emittance of subnanometer radiation can be realized; in particular, the beam size in the horizontal direction is reduced (Raimondi *et al.*, 2021 ▸). As a result, the beam shape is close to circular instead of the horizontally elongated shape obtained at the third-generation synchrotron radiation facility. This eliminates the need for the pinhole that serves as a virtual light source, thus eliminating beam loss, particularly in the horizontal direction. Nano-beam focusing of such a circular beam can yield photon fluxes three orders of magnitude higher than the conventional one. However, this requires the stability of the photon beam to be at the 10 nrad level (Yabashi & Tanaka, 2017 ▸).

Since the photon beam position is sensitive to angular fluctuations in the light source at the observation point which, in this case, is more than 30 m away from the source point, it is effective to monitor not only the electron beam itself but also the photon beam emitted from the electron beam for small angular stabilizations of the light source. Therefore, the X-ray beam monitoring system is one of the key points in DLSRs.

Although the photon beam should be monitored upstream of the optical components to avoid the influence of their instability, it is difficult to accurately observe its shape, because the undulator radiation upstream contains various photon energies, including higher-order harmonic light that is spatially distributed over a wide area, not just near the beam axis (Tanaka, 2021 ▸). Nevertheless, the X-ray beam position has been estimated by measuring the surrounding radiation without observing the beam axis itself (Aoyagi *et al.*, 2004 ▸, 2021 ▸; Shu *et al.*, 1998 ▸).

A thin diamond film has been widely used for beam diagnostics in many synchrotron radiation and X-ray free-electron laser (XFEL) facilities based on its luminescence and scattering properties including excellent heat resistance and transparency to X-rays (Tono *et al.*, 2011 ▸; Park *et al.*, 2018 ▸; van Silfhout *et al.*, 2020 ▸; Morse *et al.*, 2007 ▸). Since the XFEL has excellent monochromatic properties, it is practically sufficient to use a thin diamond film as a luminescence or scattering screen monitor without a high energy resolution to confirm the XFEL beam axis. On the other hand, when used as a monitor for undulator radiation in a ring accelerator, the profile is too flat to detect the beam centroid due to the lack of energy resolution (Kudo *et al.*, 2006 ▸). In particular, it is impossible to observe the beam axis of such a small beam exiting from the front-end using this approach.

We have shown that the photon beam centroid can be detected even upstream of a monochromator, as long as it is energy resolved, by demonstrating a pink-beam profile monitor that combines the photoluminescence of diamond with a fast-moving filter (Takahashi *et al.*, 2016 ▸). We also found that the beam shape can be observed by monitoring the X-rays scattered from the diamond film with a pinhole camera and that the image can be energy-resolved using a direct detection 2D X-ray detector (Kudo *et al.*, 2020 ▸). The undulator beam axis can be diagnosed by integrating the images of only the fundamental radiation that is discriminated using this method. Although we initially used polycrystalline diamond in the beam monitors for cost reasons, it became apparent that the image was significantly degraded because of the diffraction from the crystal grains. Moreover, single-crystal artificial diamond has become less expensive in recent years, therefore, we used a single-crystal diamond in this study.

## 2. Beam imaging method

The study was conducted at the SPring-8 beamline BL05XU, which is intended for small- and wide-angle X-ray scattering measurements using an in-vacuum undulator with a magnetic period length of 32 mm and a total length of 3 m as a light source. It is a shorter version of the SPring-8 standard in-vacuum undulator that provides horizontally polarized radiation (Hara *et al.*, 1998 ▸). A front-end (FE) slit (Oura *et al.*, 1998 ▸) is located approximately 29 m from the source point so as to finally shape the undulator radiation, namely to determine the beam size exiting the front-end. The emitted photon beam irradiates a single-crystal diamond thin film at a distance of 35.5 m from the source point, on the upstream side of a liquid-nitro­gen-cooled double-crystal monochromator (DCM).

**Figure 1:**
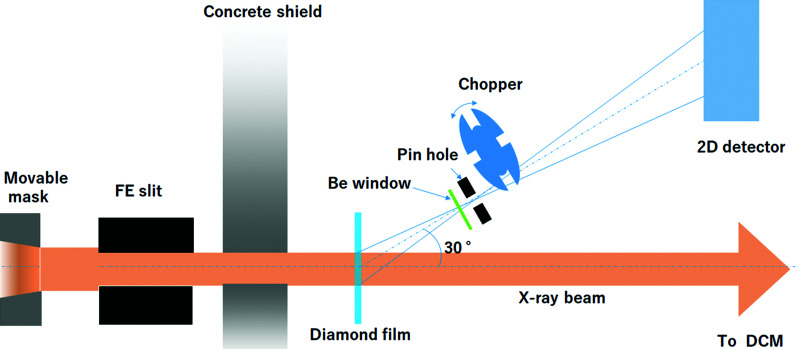
Schematic of the X-ray beam monitoring system using a pin-hole camera.

Fig. 1 ▸ shows a schematic of the X-ray beam monitoring system comprising a pinhole camera. The forward-scattered X-rays from the water-cooled diamond are imaged using the pinhole camera and then detected using a 2D detector. The diamond is placed in a vacuum chamber with a pressure in the 10^−6^ Pa range, which has a viewport in the downstream side from which the beam incidence point of the diamond can be viewed from 30° above the horizontal plane. A beryllium window with a thickness of 250 µm is attached to the viewport to emit the scattered X-rays to air. The pinhole, made of tungsten with a thickness of 500 µm and a diameter of 10 µm, is mounted on the outer side of the beryllium window.

A rotating attenuation disk made of tungsten was placed approximately 10 mm from the pinhole as a chopper so that the one-photon condition for the energy discrimination could be achieved using the 2D detector. The disk, having four slits with a width of 5 mm on the outer edge, was rotated to open at approximately 30 Hz. Since the radius of the attenuation disk is 40 mm, the exposure time of the 2D detector (25 ms per shot) can be reduced to 0.7 ms per shot. The distances from the diamond to the pinhole and from the pinhole to the detector were 194.4 mm and 400 mm, respectively.

Measurements were performed as part of the beamline adjustment period with a beam current of 10 mA, *i.e.* 1/10 of the normal use operation current (100 mA). For all experiments, the fundamental energy was set to 12.4 keV, which corresponds to an insertion-device (ID) gap of 17.26 mm. The maximum aperture size of the FE slit at BL05XU is typically limited to 1.5 mm (H) × 0.5 mm (V), which is determined based on the allowable heat load acting on the optical components under 100 mA operation and minimum ID gap. Because we were able to conduct the measurements using a beam current of 10 mA, the aperture size of the FE slit could be enlarged to 3.6 mm (H) × 2.8 mm (V), wider than the typical limit. Consequently, we were able to easily observe the spatial distribution of undulator radiation over a wide area.

### 2.1. Single-crystal diamond film

**Figure 2:**
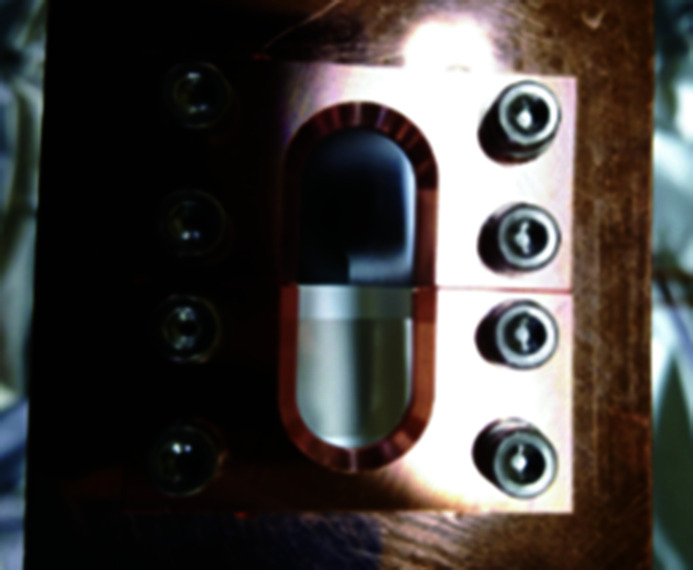
Installation of a diamond film on the copper block cooling system.

The single-crystal diamond thin film grown using the microwave plasma chemical vapor deposition into a rectangle of 15 mm × 10 mm was supplied by EDP Corporation (http://www.d-edp.jp/). One side was polished with an arithmetic average roughness (Ra) of less than 5 nm, and the other side remained as a separate surface from the parent crystal with a surface roughness of less than a few micrometres. The crystal surface is tilted 3° from the (100) plane, and no grain boundaries exist in the central area of 5 mm × 5 mm. As the thickness of the film is 70 µm, the transmittance at a photon energy of 12.4 keV is 97%. As shown in Fig. 2 ▸, two pieces of diamond are mounted on a water-cooled copper block using a holding plate independently, top and bottom. The thin film on the top side, which appears black in the image, is a polycrystalline diamond thin film (manufactured by Sumitomo Electric Industries) that we used in previous studies (Kudo *et al.*, 2020 ▸). The image also shows that the single-crystal diamond on the bottom side has a high transparency in the visible light range. Two diamonds can be selected by a stepping motor driven vertically. After the measurement, the diamonds can be completely retracted from the photon beam.

### 2.2. Detector

The detector for the pinhole camera system was SOPHIAS-L, which is a low-noise version of SOPHIAS (Hatsui & Graafsma, 2015 ▸); it is a direct-detection-type complementary metal-oxide-semiconductor image sensor (1.9 Mpixels, 30 µm square pixels, image area of 26.7 mm × 64.8 mm) manufactured by LAPIS Semiconductor Co. Ltd using the silicon-on-insulator process (Okihara *et al.*, 2012 ▸). Owing to its low noise characteristics of 67 e in r.m.s. it can be applied not only to measurements in the hard X-ray region but also in the tender X-ray region (Abe *et al.*, 2021 ▸). The sensor has integral pixels, and the detection efficiency under 12.4 keV is 86%. It was operated at 30 frames s^−1^. The exposure and readout times (dead time) of each frame were 25 ms and 8.3 ms, respectively. The acquired raw image data were processed by flat-field and dark correction. The flat-field was measured in advance using a copper-target X-ray tube (Hamamatsu Photonics K.K.,L9631-33). The point spread function (PSF) of the signal charge was measured using an optical method described elsewhere and was approximately 20 µm full width at half-maximum (FWHM; Nakajima *et al.*, 2021 ▸). Based on the proportional relationship between the signal charge and the photon energy calibrated using ^55^Fe and ^109^Cd sources, photon energy discrimination was performed using the method introduced in the next section.

### 2.3. Computer algorithm for providing energy-resolved images

**Figure 3:**
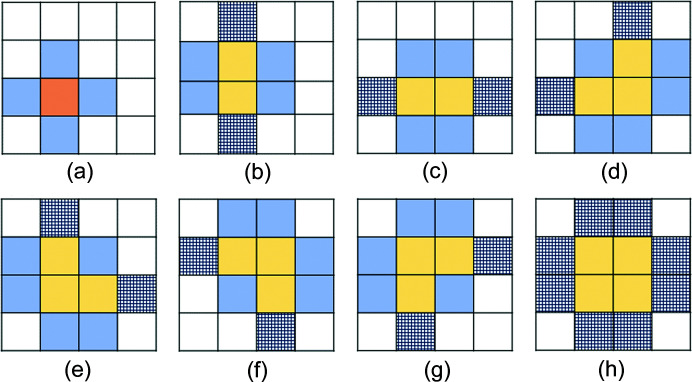
The photon signal is found by matching the detected charge shape with eight patterns (*a*)–(*h*) for each of the 4 × 4 pixels in SOPHIS-L. To identify the pattern, thresholds TH1 and TH2 are introduced. The relationship between the pixel signals is in the order of red > TH1 and yellow > TH2 > blue.

The charge generated by a single photon seeps into adjacent pixels based on the PSF of the 2D detector; therefore, we have to recover the charge amount shared by multiple pixels to measure the energy of each photon. We first found single-photon events by matching with the possible spread patterns using two thresholds: TH1 (higher threshold) and TH2 (lower threshold). The spread patterns for matching the single-photon signal were determined by referring to a previous article (Blaj *et al.*, 2019 ▸), as shown in Fig. 3 ▸. TH1 is used to find the pattern shown in Fig. 3 ▸(*a*), and TH2 is used to find patterns Figs. 3 ▸(*b*) to 3(*h*). The photon signal is concentrated at one pixel in pattern (*a*) but spreads to adjacent pixels in patterns (*b*) to (*h*). The photon position was assigned to the pixel with the maximum charge. The charge amount was recovered by summing the charges for the pixels in red and yellow discriminated by TH1 and TH2. To improve the resolution in the reconstructed photon energy, we further summed a portion of the blue pixels (excluding the mesh pattern), having a charge greater than the average of all the blue pixels. In the cases of (*b*) and (*c*), only the pixels on both sides of the pixel with the maximum value were targeted. The thresholds were optimized as TH1 = 80 ADU and TH2 = 40 ADU to increase the efficiency and reduce the fake rate in the photon energy range between 10 keV and 30 keV, which is the target in this measurement [*cf*. photon charge is 125 ADU for 5.9 keV (^55^Fe) in the SOPHIAS-L detector system].

## 3. Beam image measurement

### 3.1. Improvement of image quality using single-crystal diamond film

**Figure 4:**
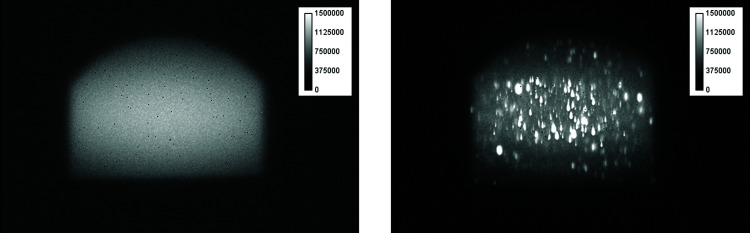
Beam images for a fundamental energy of 12.4 keV captured using a single-crystal diamond film (left) and a polycrystalline diamond film (right).

Fig. 4 ▸ shows a simple integrated beam image with a single-crystal diamond film (left) and with a polycrystalline diamond thin film (right). These images were taken without the attenuation rotary disk. In the case of the polycrystalline diamond, as shown in Fig. 4 ▸ (right), diffraction is observed in a wide area, including at the center of the beam, whereas in the case of the single-crystal diamond (left), the diffraction spots disappear. The upper part of the image is cut in the form of a circle, which is the shadow of the movable mask located upstream of the FE slit. After capturing these images, the movable mask was moved by 0.6 mm, in which case the FE-slit aperture appears to be rectangular. The image of the beam shown in Fig. 4 ▸ (left) is still flat even though the diffraction spot has disappeared, and it is difficult to determine the center of the beam without applying the energy discrimination presented in the next section.

### 3.2. Energy-resolved beam images by droplet analysis

**Figure 5:**
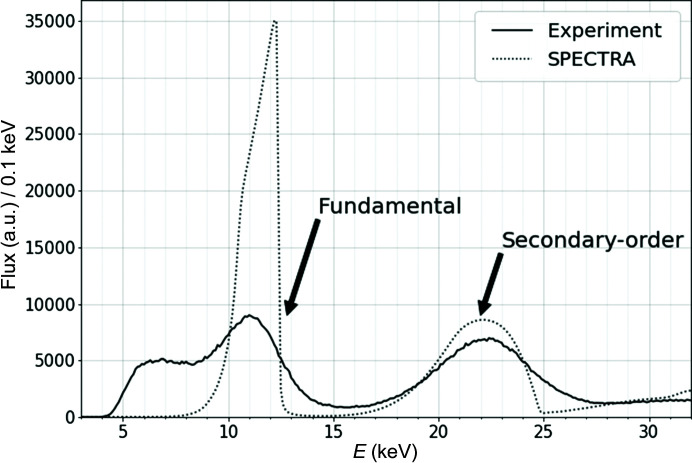
Photon energy spectrum for a fundamental energy of 12.4 keV. Data within a 1 mm square in both the horizontal and the vertical directions from the beam center are selected. The solid line represents the spectrum for the experiment after the droplet analysis. The dotted line represents the spectrum calculated using *SPECTRA*.

Using the droplet analysis described in Section 2.3, we obtained a photon beam spectrum from 10 000 images of the pinhole camera. Fig. 5 ▸ shows the photon beam energy spectrum after the droplet analysis (solid line) when the fundamental energy is 12.4 keV, along with the calculation result (dashed line) obtained using the synchrotron radiation calculation code *SPECTRA* (Tanaka, 2021 ▸) which has an asymmetric shape. The horizontal axis was calibrated using ^55^Fe and ^109^Cd sources. When measured using a 2D detector, the shape is convoluted with a response function of the 2D detector. Thus, the measured spectrum is smoothed, and the peak position is shifted to the low-energy side. On the other hand, the number of signals in the fundamental energy spectrum is less than that predicted by *SPECTRA*. This is because the photon signal in the fundamental energy spectrum could not be fully reconstructed. Patterns other than those shown in Fig. 3 ▸ were deleted as per the algorithm described in Section 2.3 due to the high occupancy of the fundamental photon.

**Figure 6:**
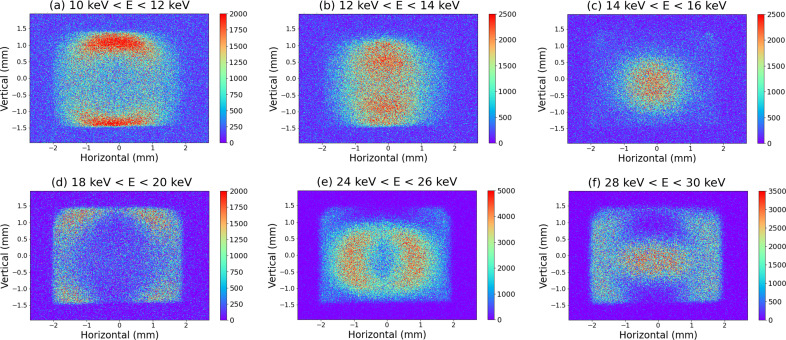
Energy-resolved beam images for a fundamental energy of 12.4 keV.

Fig. 6 ▸ shows the results of the energy discrimination by droplet analysis from 10 000 images, namely for photon energies ranging from (*a*) 10 keV to 12 keV, (*b*) 12 keV to 14 keV, (*c*) 14 keV to 16 keV, (*d*) 18 keV to 20 keV, (*e*) 24 keV to 26 keV and (*f*) 28 keV to 30 keV, at an ID gap of 17.26 mm, corresponding to a fundamental photon energy of 12.4 keV. For these data, the attenuation disk was rotated to satisfy the one-photon condition (1 photon per shot per pixel) that allows droplet analysis. Subsequently, the data when the disk was stopped in the closed status were subtracted as the background.

As shown in Fig. 6 ▸(*a*), the discriminated low-energy photons appear to be distributed separately in the upper and lower parts of the image. As the energy of the discriminated photons increases, the separated photons start to accumulate at the center, and finally, as shown in Fig. 6 ▸(*c*), the peaks seem to be at the center in both the horizontal and the vertical directions. When discriminated with even higher energies, the photons began to separate into vertical and horizontal regions [Fig. 6 ▸(*d*)]. Subsequently, as shown in Fig. 6 ▸(*f*), the photons discriminated at twice the energy shown in Fig. 6 ▸(*c*) accumulate again at the center, although it is not as clear as in Fig. 6 ▸(*c*), with a faint distribution in the surroundings.

In the configuration of this system, images through the pinhole can be attributed to both elastic and Compton scattering. Since the forward scattering from the diamond is observed from 30° upward, the energy shift due to the Compton effect is estimated to range from approximately −40 eV to −230 eV at 12 keV to 30 keV, which is low enough compared with the energy resolution of SOPHIAS-L (2 keV) in FWHM. Therefore, Compton scattering does not significantly affect the primary spectrum at a fundamental energy of 12.4 keV when observed with SOPHIAS-L. As such, the shift in the peak energy to the low-energy side shown in Fig. 5 ▸ can be attributed to other causes. When measured by a 2D detector, the spectrum shape is convoluted with a response function of the 2D detector. Subsequently, the measured spectrum is smoothed, and the peak position is shifted to the low-energy side, particularly for the undulator spectrum with an asymmetric shape.

**Figure 7:**
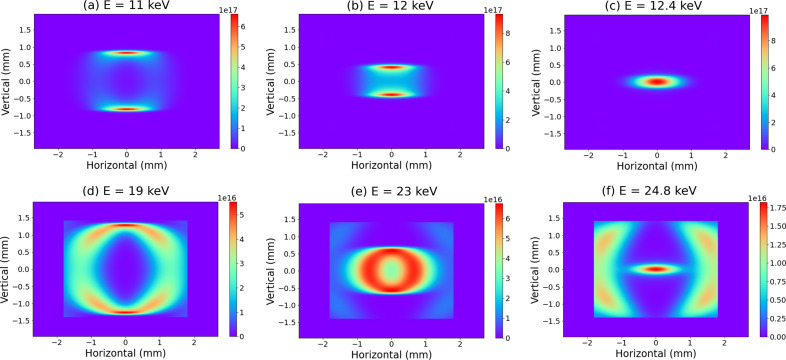
Beam images calculated using *SPECTRA* for a fundamental energy of 12.4 keV with BL05XU undulator parameters. The simulation areas were limited to within 3.6 mm (H) and 2.8 mm (V).

Fig. 7 ▸ shows the beam shape at each photon energy calculated using *SPECTRA*, in which the energy bandwidth is 0.1% by default. Fig. 7 ▸(*c*) shows the photon distribution of the fundamental radiation peak, which is concentrated at the center. On the other hand, Figs. 7 ▸(*a*) and 7(*b*) show low-energy components of the fundamental peak, and the photon distribution fluctuates. Similarly, Fig. 7 ▸(*f*) shows the peak of the second-order harmonic radiation, and Figs. 7 ▸(*d*) and 7(*e*) show the low-energy components of the second-order harmonic. The images obtained by the pin-hole camera system, shown in Fig. 6 ▸, are in agreement with that calculated using *SPECTRA*. The energy from the *SPECTRA* calculation (Fig. 7 ▸) does not exactly match the energy range (Fig. 6 ▸) discriminated for the droplet analysis. These shifts can be attributed to the convolution of the asymmetric ID spectrum and the energy resolution characteristics of SOPHIAS-L. Thus, the beam axis of the fundamental radiation can be detected from the discrimination in the energy range 14–16 keV.

**Figure 8:**
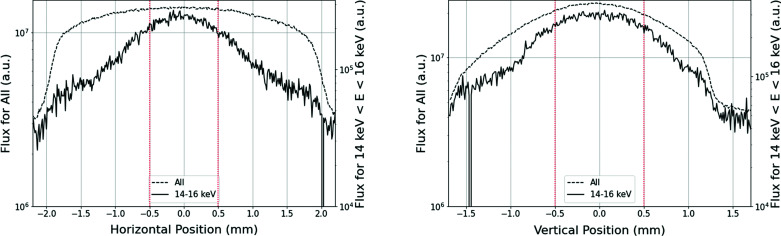
Horizontal (left) and vertical (right) beam profiles with all energy ranges (dashed line) and with 14 keV < *E* < 16 keV (solid line) for a fundamental energy of 12.4 keV.

Fig. 8 ▸ shows the beam profiles in the horizontal (left) and the vertical (right) directions for photon energies ranging from 14 keV to 16 keV (solid line). Compared with the beam profile obtained from the simple integrated image shown in Fig. 4 ▸(*a*) (dashed line), we find that the energy discrimination clearly sharpens the profile, particularly in the horizontal direction. As for the vertical direction, the energy discrimination is less effective. The area surrounded by the red dotted line corresponds to the FE slit aperture size of 1 mm × 1 mm, demonstrating that it is possible to calculate the beam centroid from this aperture both horizontally and vertically.

## 4. Conclusions

We found that a single-crystal diamond film can effectively reduce contamination due to the diffraction from crystal grains, which has been a major problem when using a polycrystalline diamond film. As a result, we could capture much better beam images than before, and at a fundamental energy of 12.4 keV, which is the practical beam energy, the many diffraction spots disappeared; thus, we obtained various energy-resolved images consistent with the *SPECTRA* calculation. On the other hand, in the case of single crystals, although there are not as many diffraction spots as in the case of the polycrystalline diamond, there is intense diffraction in the direction where a few Bragg conditions are satisfied. If the diffraction spots match with the pinhole direction, it is expected that the beam is strong enough to damage the detector, and the beam image could not be appropriately obtained. In advance, a simulation was performed along the diffraction direction in the Laue case that can occur from the arrangement of the single crystal in this study. It was confirmed that higher-order reflections near the pinhole direction are possible, but perfect matching is rare. Moreover, in this experiment, it was confirmed that no significant diffraction through the pinhole appeared for ID gaps ranging from 17.26 mm to fully open (fundamental energy range 12.4–19 keV).

In this measurement, the exposure time of SOPHIAS-L was decreased to 1/35 using the rotating attenuation disk, and furthermore, it was conducted at a beam current of 10 mA, which is 1/10 of the ring current in the user time. These procedures were necessary to establish a single-photon condition to enable energy discrimination using the droplet analysis. Under these conditions, it took approximately 330 s to acquire 10 000 images for the energy discrimination with SOPHIAS-L at a frame rate of 30 frames s^−1^. If we introduce a detector with a higher frame rate and shorter exposure time, we can achieve a measurement with a stored beam current of 100 mA and without a rotating disk; this can reduce the measurement time by two orders of magnitude to a few seconds for the same measurement. As for the data processing time, it is time consuming because the droplet analysis needs to be performed on an offline computer. To realize real-time performance, an on-board analysis using a field-programmable gate array (FPGA) could be effective, in which a detector with a small PSF could be used, and a simple threshold discrimination technique without droplet processing could be implemented.

By applying energy discrimination to the images, which were qualitatively improved using a single-crystal diamond, the radiation pattern for each photon energy, corresponding to the *SPECTRA* calculation, could be observed without passing through the monochromator. For viewing the entire radiation pattern, we used an FE slit 3.6 mm (H) × 2.8 mm (V) in size for this measurement, though it is not practical for 100 mA operation from the viewpoint of heat resistance of the optical components. Thus, to contribute to the stabilization of the light source, this system should be operated with a narrower FE slit so that the heat load acting on the optical components, such as the monochromator, could be reduced to a safe value even under a further narrow ID gap condition with 100 mA operation. Judging from the beam profile obtained by energy discrimination, the FE slit aperture size of 1 mm × 1 mm would be sufficient to detect the centroid of the X-ray beam. To improve the performance of this system, a detector with a higher energy resolution, frame rate and shorter exposure time is necessary. It can provide sharper energy-resolved images in a short time and is expected to decrease the aperture size requirement of the FE slit.
